# Supplementary figures and images for: Hypoxia Triggers TAZ Phosphorylation in Basal A Triple Negative Breast Cancer Cells
Source: Int J Mol Sci. 2022 Sep 4;23(17):10119. doi: 10.3390/ijms231710119 (PMC9456181; doi:10.3390/ijms231710119)

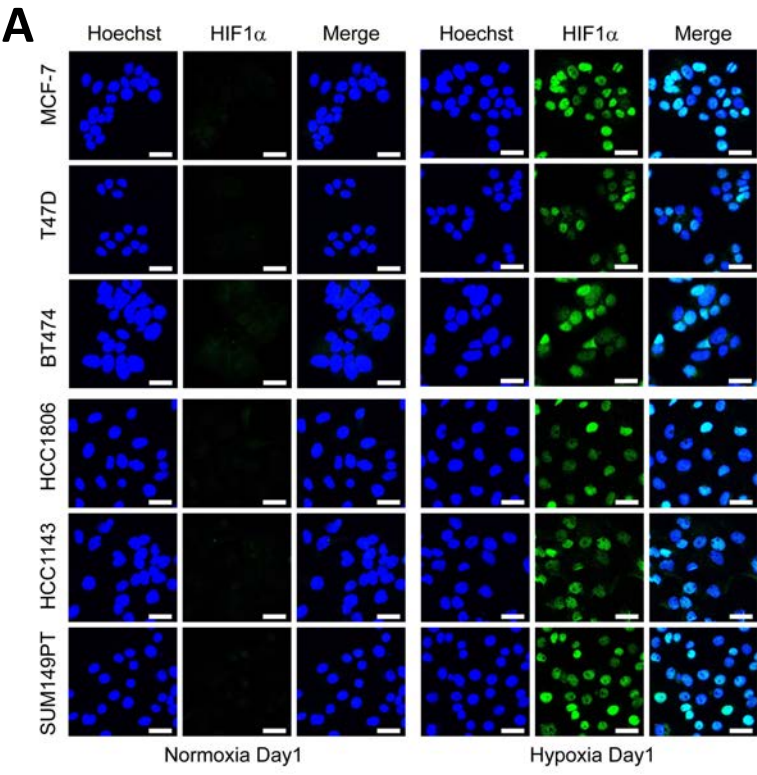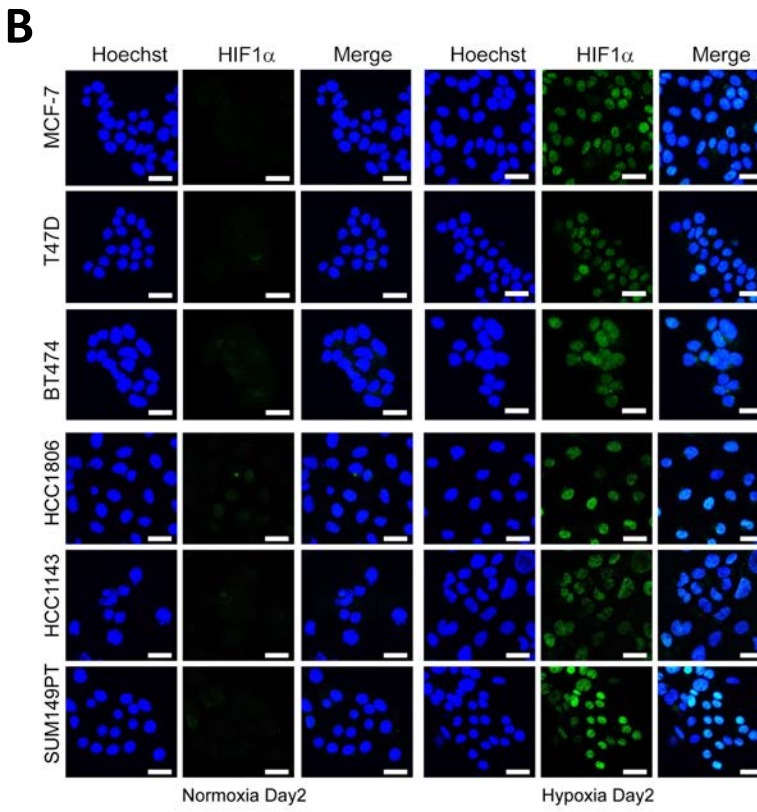

# MCF-7: GSEA all DEGs

A

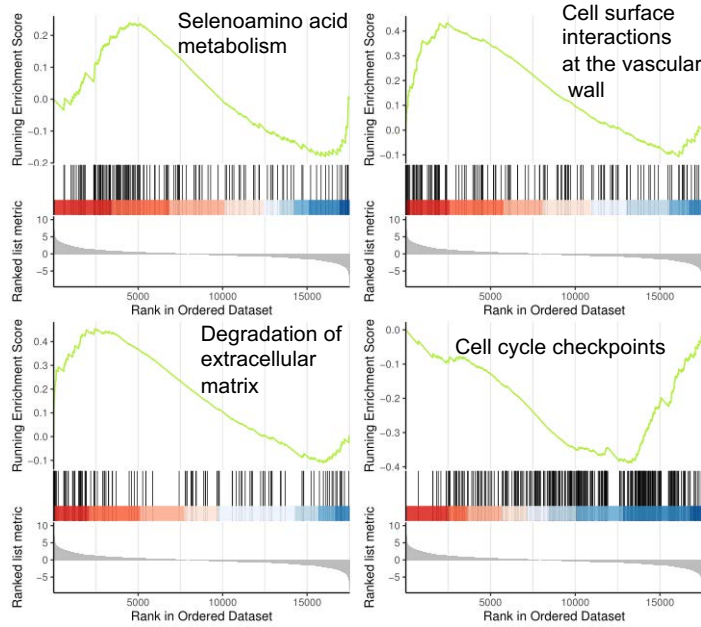

B

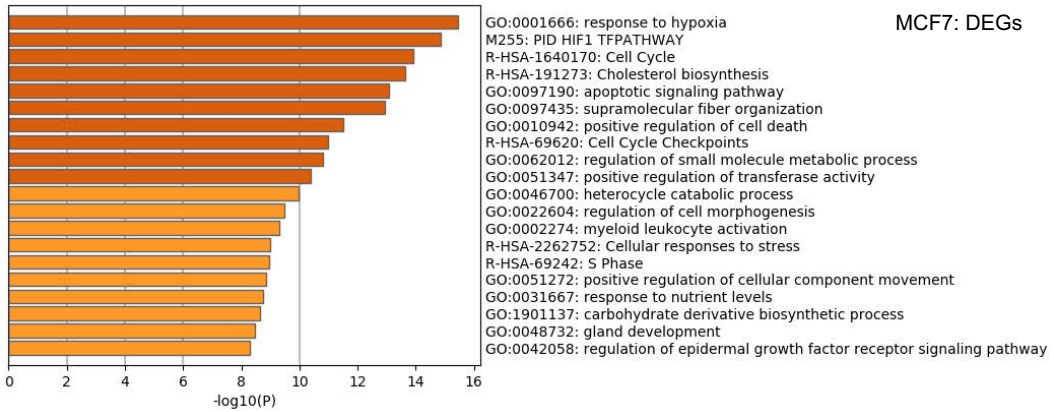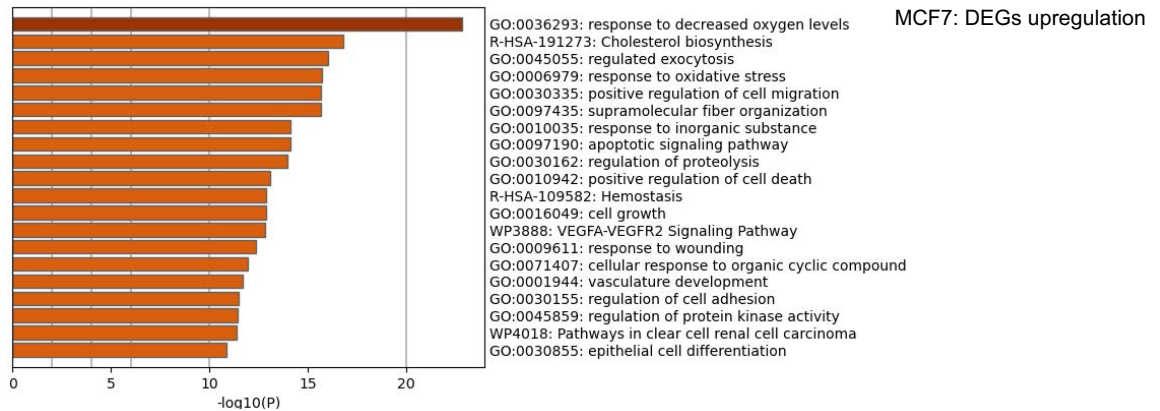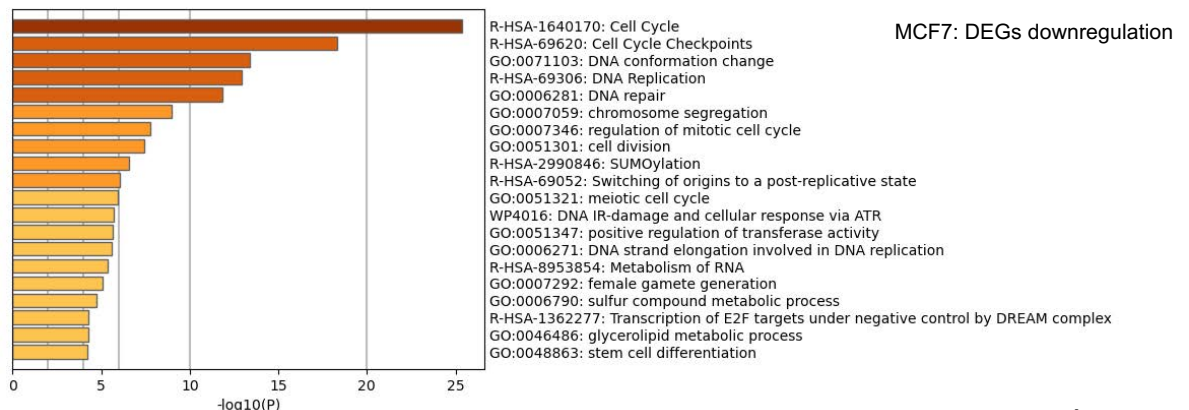

A

HCC1143: GSEA all DEGs

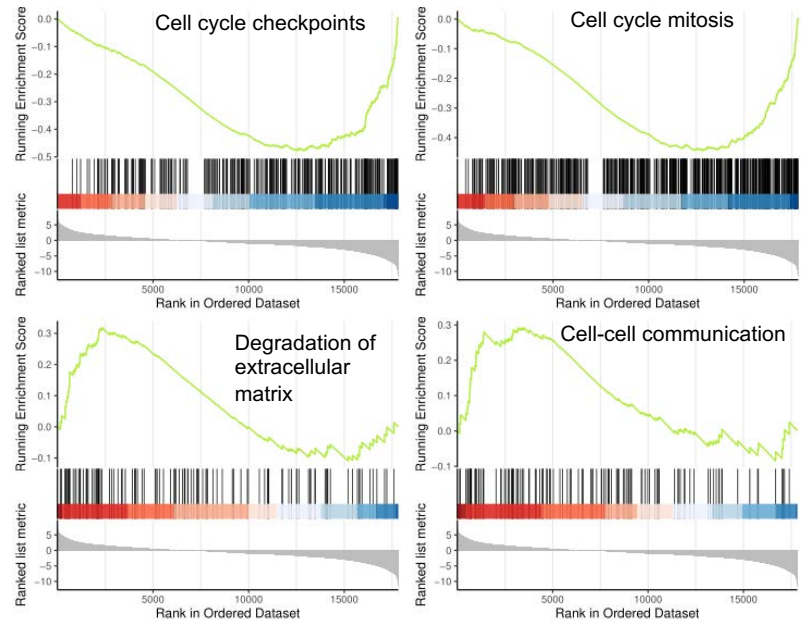

B

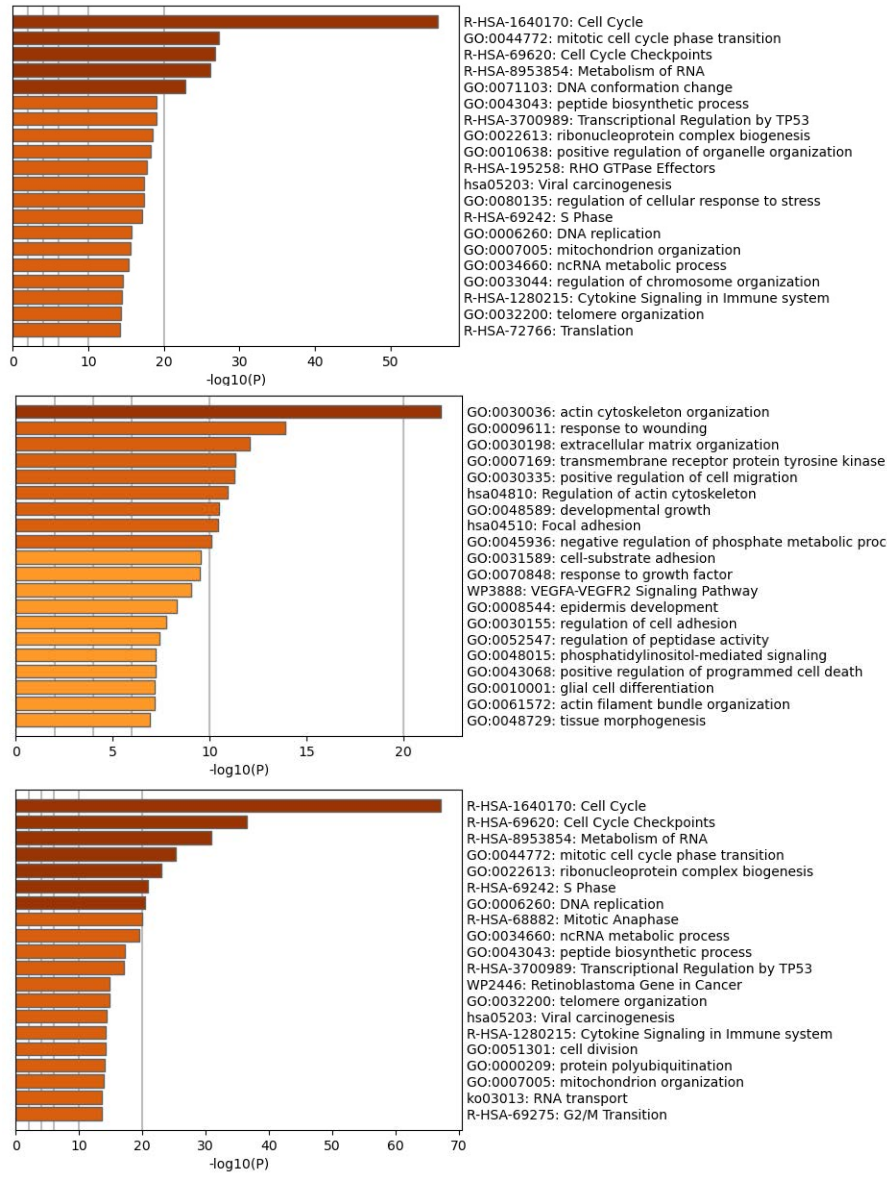

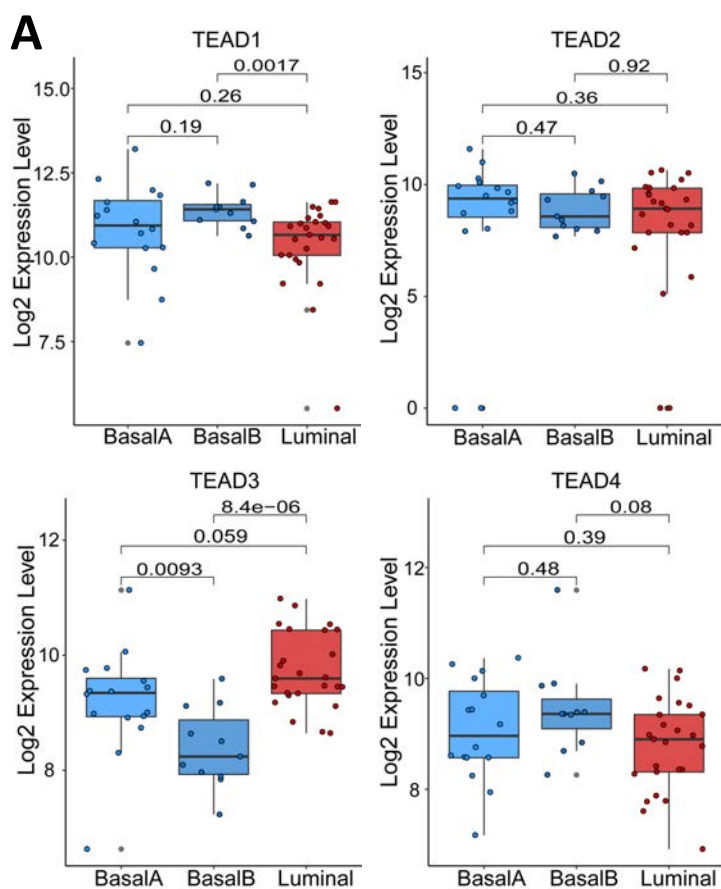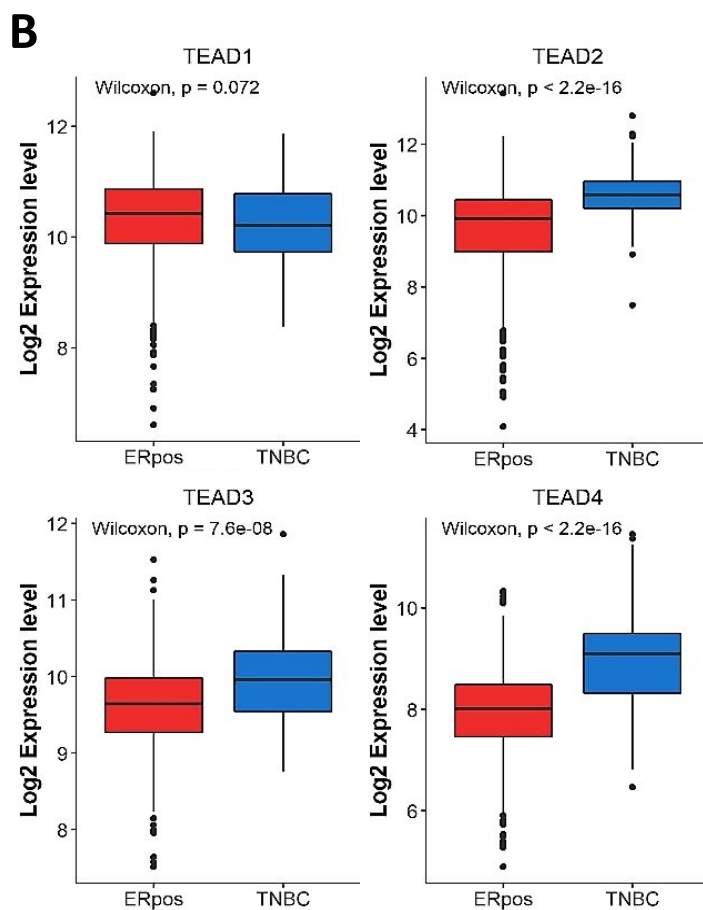

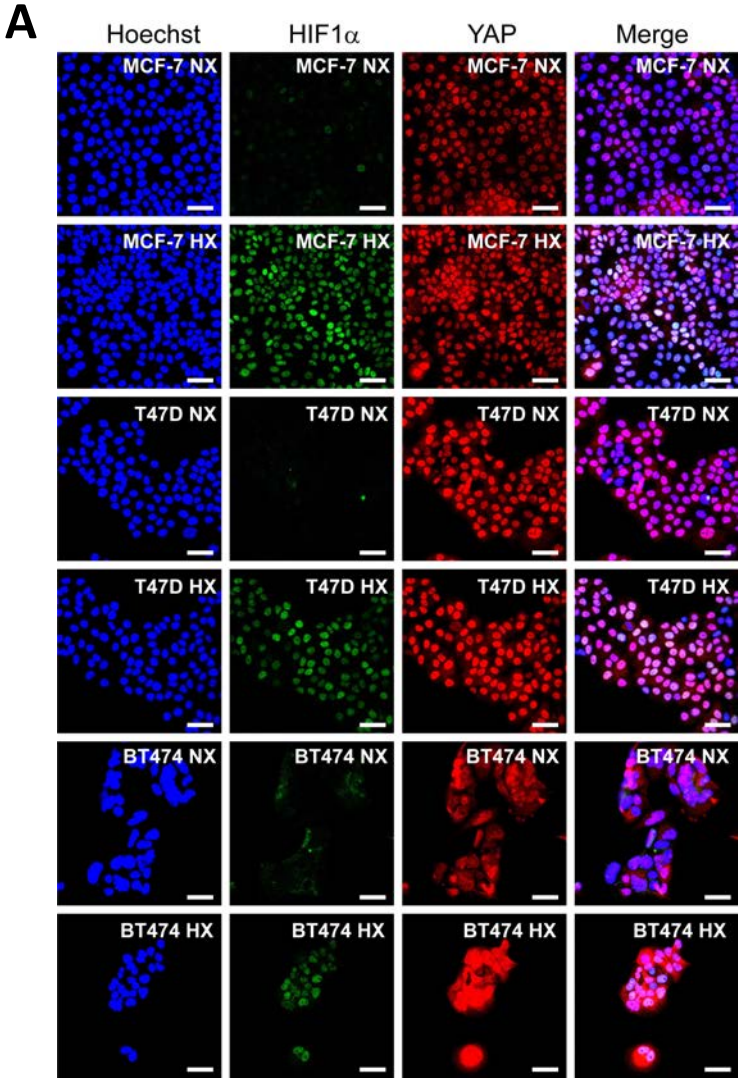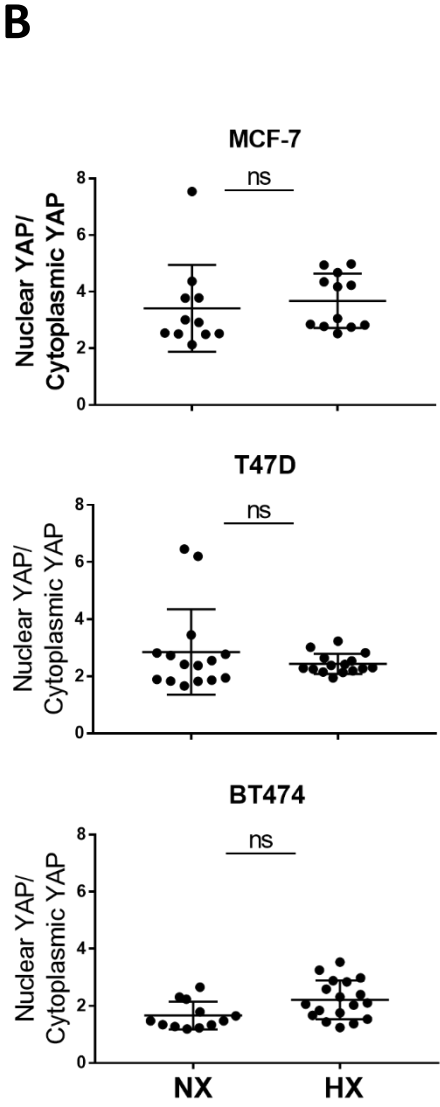

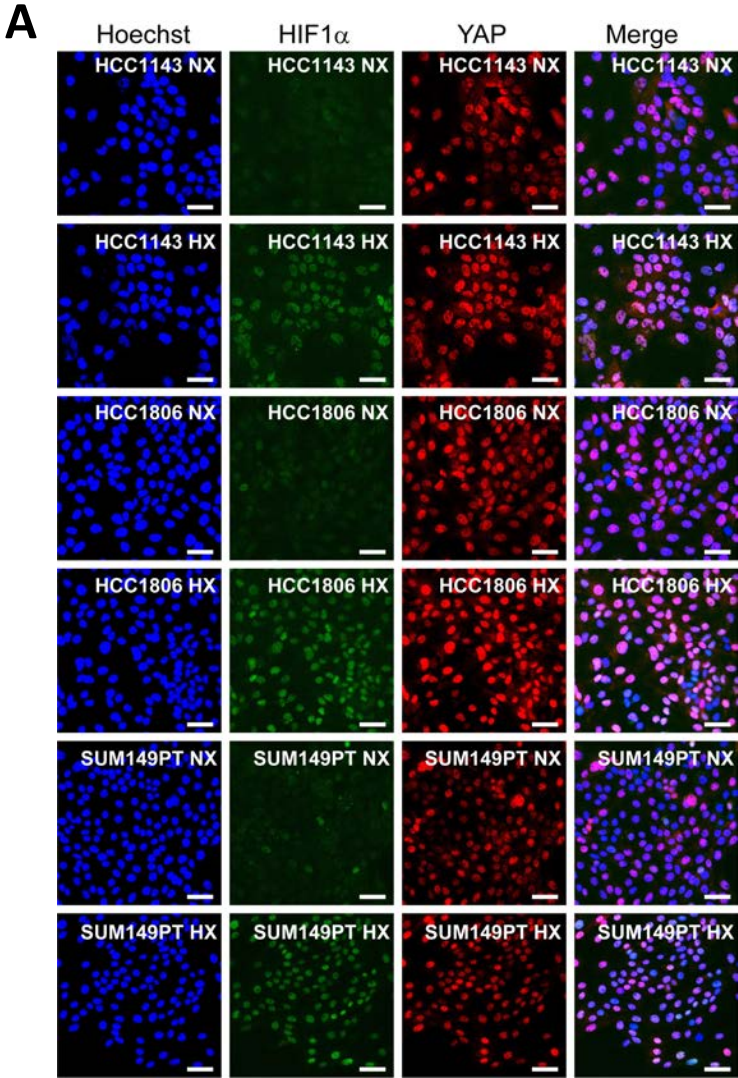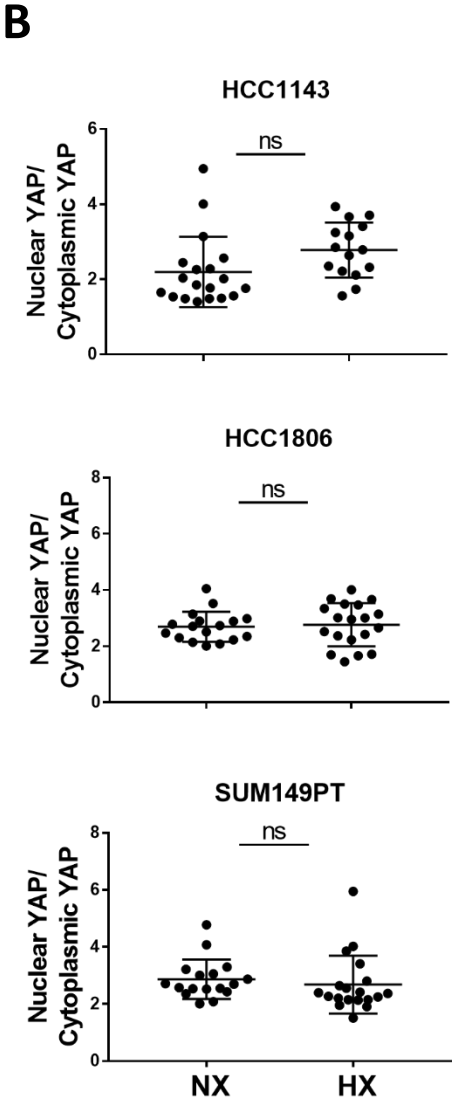

Supplement: Supplementary file 1 [file ijms-23-10119-s001.zip › supplementary materials/Qiuyu et al 2022 suppl figures.pdf]
